# The Plasma Membrane Purinoreceptor P2K1/DORN1 Is Essential in Stomatal Closure Evoked by Extracellular Diadenosine Tetraphosphate (Ap_4_A) in *Arabidopsis thaliana*

**DOI:** 10.3390/ijms242316688

**Published:** 2023-11-24

**Authors:** Jędrzej Dobrogojski, Van Hai Nguyen, Joanna Kowalska, Sławomir Borek, Małgorzata Pietrowska-Borek

**Affiliations:** 1 Department of Biochemistry and Biotechnology, Faculty of Agriculture, Horticulture and Bioengineering, Poznań University of Life Sciences, Dojazd 11, 60-632 Poznań, Poland; jedrzej.dobrogojski@up.poznan.pl; 2 Division of Biophysics, Institute of Experimental Physics, Faculty of Physics, University of Warsaw, Pasteura 5, 02-093 Warsaw, Poland; nguyen.van-hai@fuw.edu.pl (V.H.N.); joanna.kowalska@fuw.edu.pl (J.K.); 3 Department of Plant Physiology, Faculty of Biology, Adam Mickiewicz University Poznań, Uniwersytetu Poznańskiego 6, 61-614 Poznań, Poland; borek@amu.edu.pl

**Keywords:** abscisic acid, diadenosine tetraphosphate (Ap_4_A), dicytidine tetraphosphate (Cp_4_C), dinucleoside polyphosphates (Np_n_Ns), extracellular ATP (eATP), plant signalling, reactive oxygen species (ROS), uncommon nucleotides

## Abstract

Dinucleoside polyphosphates (Np_n_Ns) are considered novel signalling molecules involved in the induction of plant defence mechanisms. However, Np_n_N signal recognition and transduction are still enigmatic. Therefore, the aim of our research was the identification of the Np_n_N receptor and signal transduction pathways evoked by these nucleotides. Earlier, we proved that purine and pyrimidine Np_n_Ns differentially affect the phenylpropanoid pathway in *Vitis vinifera* suspension-cultured cells. Here, we report, for the first time, that both diadenosine tetraphosphate (Ap_4_A) and dicytidine tetraphosphate (Cp_4_C)-induced stomatal closure in *Arabidopsis thaliana*. Moreover, we showed that plasma membrane purinoreceptor P2K1/DORN1 (does not respond to nucleotide 1) is essential for Ap_4_A-induced stomata movements but not for Cp_4_C. Wild-type Col-0 and the *dorn1-3 A. thaliana* knockout mutant were used. Examination of the leaf epidermis *dorn1-3* mutant provided evidence that P2K1/DORN1 is a part of the signal transduction pathway in stomatal closure evoked by extracellular Ap_4_A but not by Cp_4_C. Reactive oxygen species (ROS) are involved in signal transduction caused by Ap_4_A and Cp_4_C, leading to stomatal closure. Ap_4_A induced and Cp_4_C suppressed the transcriptional response in wild-type plants. Moreover, in *dorn1-3* leaves, the effect of Ap_4_A on gene expression was impaired. The interaction between P2K1/DORN1 and Ap_4_A leads to changes in the transcription of signalling hubs in signal transduction pathways.

## 1. Introduction

Regulation of plant metabolic processes takes place at a molecular level. The defence reactions are among the processes in which signal transduction plays a key role. Based on the criterion of the distance that a given signal molecule can cover, short-distance molecules cause local intercellular responses, and long-distance molecules trigger systemic responses. Signalling molecules regulate many processes throughout various signal transduction pathways and specific or unspecific receptors [1]. Unlike animals, the ability of extracellular nucleotides to initiate diverse signalling responses in plants remained enigmatic for years. A growing number of nucleotides classified as signalling molecules have been identified in plants [2]. Among them, extracellular ATP (eATP) plays an essential role in plant growth [3,4,5,6,7,8] and development [9,10]. Extracellular ATP regulates responses to biotic stress [11,12,13,14] and abiotic stress [15,16,17,18]. One of the reactions that eATP can control is stomatal movements [12,19,20]. In this reaction, the cytoplasmic Ca^2+^ ions ([Ca^2+^]_cyt_) and the complex signalling cross-talk between second messengers, such as nitric oxide (NO) [7,21,22], and reactive oxygen species (ROS) [12,23,24,25] plays a crucial role as a mediator in the signal transduction pathway. Consequently, these messenger agents affect the phosphorylation of mitogen-activated protein kinase (MAPK) and the expression of defence-related genes [12,26,27].

We have a longstanding interest in the function of dinucleoside polyphosphates (Np_n_Ns) in plant cells. Our papers describe changes in gene expression profile and metabolism in *Arabidopsis thaliana* and *Vitis vinifera* treated with a broad spectrum of Np_n_Ns. We postulated the participation of Np_n_Ns in the plant defence responses since they induce synthesis of the phenylpropanoid pathway-delivered secondary metabolites [28,29,30]. The phenylpropanoid pathway participates in plant defence responses [31,32]. Identification of Ap_4_A and other Np_n_Ns across prokaryotic and eukaryotic cells testifies to their universality [33]. Due to the dramatic increase in levels of various Np_n_Ns observed in cells subjected to abiotic stress factors [34,35,36,37,38], these compounds have been termed “alarmones”, triggering stress adaptive processes. Our latest findings confirmed the induction of the phenylpropanoid pathway by purine, pyrimidine, and purine–pyrimidine hybrids of Np_n_Ns. Moreover, we observed that diadenosine polyphosphates (Ap_n_A) induced stilbene biosynthesis. In contrast, dicytidine polyphosphates (Cp_n_C) strongly inhibited this reaction but markedly induced the expression of the cinnamoyl-CoA reductase gene that controls lignin biosynthesis [30]. Nonetheless, the underlying mechanism of Np_n_N signal recognition and transduction in plants remains elusive. The growing number of plant enzymes found to be involved in Np_n_N biosynthesis and degradation strengthens the hypothesis of their signalling function [2,33].

Plants can respond to extracellular purine nucleotides, such as eATP, through plasma membrane receptors. So far, two plant receptors with an eATP binding domain have been identified. They are P2K1/DORN1 (does not respond to nucleotides 1) [26] and P2K2/DORN2, which belong to the L-type lectin receptor kinase (LecRK) protein family [39,40]. LecRK proteins activate the processes controlling stress responses, development, growth, and disease resistance [41]. Although eATP sensing and action in plants have been elucidated, the mechanisms of signal perception and transduction evoked by Np_n_Ns, such as Ap_4_A and Cp_4_C, remain enigmatic. In animal cells, among the different nucleotides and nucleosides, eATP, together with Ap_4_A, shares access to the same receptors that belong to the P2 group, which is divided into two classes, namely ligand-gated ion channels (P2Xs) and G protein-coupled (P2Ys) receptors [42,43,44,45,46]. Therefore, we hypothesise that the purinoreceptor P2K1/DORN1, a receptor of eATP, is also necessary for sensing Ap_4_A in plant cells. Moreover, we wondered whether P2K1/DORN1 is also engaged in the effects evoked by the pyrimidine nucleotide Cp_4_C.

Here, we present, for the first time, evidence for the involvement of the P2K1/DORN1 receptor in the sensing of Ap_4_A in plants. All experiments were conducted on 4-week-old *Arabidopsis thaliana* wild-type Col-0 and *dorn1-3* knockout mutant leaves. Our research showed that extracellular Ap_4_A and Cp_4_C evoked stomatal closure in Col-0 plants. This effect was abolished in the *dorn1-3* mutant by Ap_4_A but not Cp_4_C. This result confirms the requirement of P2K1/DORN1 for Ap_4_A-induced stomatal closure. Nevertheless, our research indicates the involvement of superoxide (^•^O_2_^−^) and hydrogen peroxide (H_2_O_2_) in the signal transduction evoked by Ap_4_A and Cp_4_C, leading to stomatal closure. Furthermore, we analysed the expression of genes encoding selected proteins integrated within the signalling hubs. It concerns NADPH oxidases (*RBOHD* and *RBOHF*), *MAPK* cascades, *SNF1/AMPK*-related protein kinases (*SnRKs*), and transcriptional factors, such as *ZAT6* and *ZAT12*. Notably, Ap_4_A induced the expression of the tested genes. Moreover, the gene expression in *dorn1-3* was almost abolished by the Ap_4_A effect.

## 2. Results

### 2.1. Ap_4_A and Cp_4_C Induce Stomatal Closure

**Figure 1:**
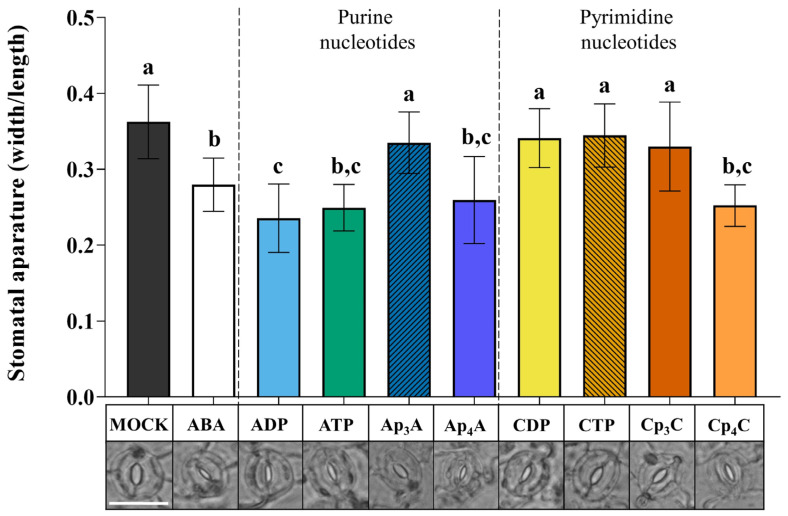
Diadenosine tetraphosphate (Ap_4_A) and dicytidine tetraphosphate (Cp_4_C), similar to adenosine diphosphate (ADP) and adenosine triphosphate (ATP), induce stomatal closure in *Arabidopsis thaliana* Col-0 plants. Images represent stomata in the abaxial epidermis of a leaf treated for 2 h with the MOCK solution MES/KOH opening buffer, 10 µM abscisic acid (ABA), and 2 mM purine and pyrimidine nucleotides. White bar = 25 μm. Bars represent mean values ± SD, *n* ≥ 20, three biological replicates. Different letters above the error bars indicate statistically significant differences according to the ANOVA analysis with the post-hoc Tukey’s HSD multiple comparisons test (*p* < 0.05).

Our previous research showed that exogenous Np_n_Ns induce the biosynthesis of secondary metabolites that play an essential role in the plant defence strategy [28,29,30]. We wondered how the signal evoked by Np_n_Ns could be sensed and transduced in plant cells and whether plants contain cell membrane receptor(s) for these molecules. It is known that eATP, one of the exogenous purine nucleotides, evokes stomatal closure with the involvement of the purinoreceptor P2K1/DORN1 in *Arabidopsis thaliana* [12,26]. Therefore, based on similarities in the ATP and Ap_4_A structures, we tested the effect of these nucleotides on stomatal movements. Moreover, we also included cytidine nucleotides in our research because of the different effects of purine and pyrimidine Np_n_Ns on the phenylpropanoid pathway in *Vitis vinifera* cells [30]. To trace stomatal movement under the nucleotide treatment, we examined the ability of purine Np_n_Ns such as Ap_3_A and Ap_4_A to stimulate stomatal closure. Additionally, for the positive control, we tested the effects of ADP and ATP, as described earlier [12,26], as well as ABA—a well-known molecule controlling stomatal movements [47,48]. Exogenous Ap_4_A significantly reduced the stomatal aperture in the light. It was at a similar level compared to the effect evoked by ATP and ADP. However, Ap_3_A did not evoke such an effect (Figure 1). We also examined stomatal movement under the treatment of cytidine mono- and dinucleotides (CDP, CTP, Cp_3_C, Cp_4_C). Interestingly, only Cp_4_C triggered significant stomatal closure among tested cytidine nucleotides. As expected, ABA closed stomata [49] (Figure 1).

In plant cells, there are enzymes degrading Np_n_Ns to mononucleotides [50]. To confirm that Ap_4_A and Cp_4_C evoke stomatal closure but not by the products of their degradation (AMP, ADP, ATP, and CMP, CDP, CTP, respectively), we collected samples of leaf epidermis from the microscope slides after incubation of nucleotides, and application of the HPLC assay (Method S2) proved that Ap_4_A was not degraded to the corresponding mononucleotide. Only a trace amount of CTP was detected in a solution of Cp_4_C after the investigation (Figure S1).

### 2.2. P2K1/DORN1 Is Involved in Signal Perception Evoked by Ap_4_A but Not Cp_4_C

**Figure 2:**
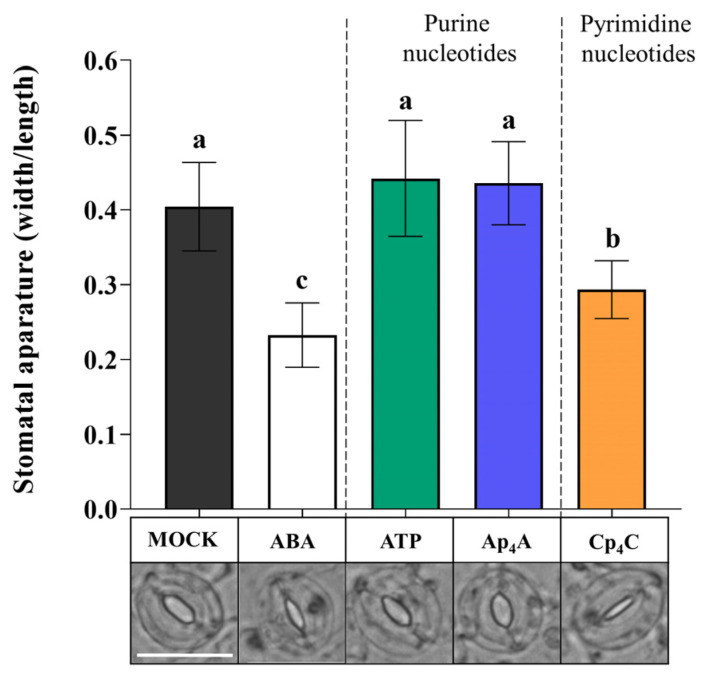
Diadenosine tetraphosphate (Ap_4_A), similar to extracellular (eATP), did not induce stomatal closure in the *dorn1-3 Arabidopsis thaliana* mutant. However, dicytidine tetraphosphate (Cp_4_C) and abscisic acid (ABA) evoked stomatal closing. Images represent stomata in the abaxial epidermis of *dorn1-3* leaf treated for 2 h with MOCK solution opening buffer, 10 µM ABA and 2 mM adenosine triphosphate (ATP), Ap_4_A, and Cp_4_C. White bar = 25 μm. Bars represent mean values ± SD, *n* ≥ 20, three biological replicates. Different letters above the error bars indicate statistically significant differences according to the ANOVA analysis with the post-hoc Tukey’s HSD multiple comparisons test (*p* < 0.05).

Plants respond to eATP by the induction of a complex signalling network after signal recognition by the P2K1/DORN1 and P2K2 receptors [26,39]. Similarities in stomatal movements evoked by eATP, Ap_4_A, and Cp_4_C led us to hypothesise that those nucleotides could interact with P2K1/DORN1. Based on the results presented in Figure 1, Ap_4_A and Cp_4_C were chosen for further experiments. The *dorn1-3* mutant, having a T-DNA insertion in the extracellular legume-type lectin domain, was selected based on literature data [12,26]. We found that Ap_4_A and eATP did not close stomata in *dorn1-3* mutant leaves. Contrary to this, Cp_4_C significantly closed stomata in *dorn1-3* mutant leaves. As expected, ABA-treated mutant leaves also showed closed stomata [12] (Figure 2). Thus, the results strongly suggest that besides eATP, P2K1/DORN1 may also be involved in signal perception elicited by Ap_4_A but not Cp_4_C.

### 2.3. ROS Are Produced in Leaves under Nucleotide Treatment

**Figure 3:**
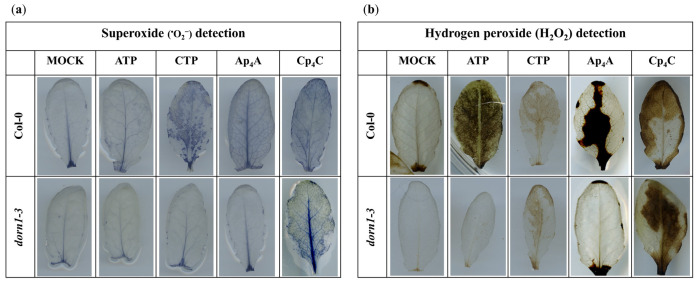
Histochemical detection of ^•^O_2_^−^ (**a**) and H_2_O_2_ (**b**) in leaves of *Arabidopsis thaliana* Col-0 and the *dorn1-3* mutant triggered by 2 mM adenosine triphosphate (ATP), cytidine triphosphate (CTP), diadenosine tetraphosphate (Ap_4_A), dicytidine tetraphosphate (Cp_4_C) after 2 h treatment. Leaves were stained with nitroblue tetrazolium (NBT) and 3,3′-diaminobenzidine tetrahydrochloride (DAB) for ^•^O_2_^−^ and H_2_O_2_ detection, respectively. The experiment was repeated six times, and representative leaves were chosen.

It was previously found that the elevated production of ROS and stomatal closure are mediated by eATP recognition by the receptor P2K1/DORN1, followed by direct phosphorylation of the NADPH oxidase RBOHD [12]. This phosphorylation causes an increase in the generation of extracellular ROS, such as ^•^O_2_^−^, which is then converted into H_2_O_2_ in the extracellular environment [51,52]. Notably, the apoplastic production of ROS is one of the fastest physiologically common responses to external stimuli observed in plants [53,54]. Considering all the above-described information, we decided to investigate the accumulation of ^•^O_2_^−^ and H_2_O_2_ in *Arabidopsis thaliana* leaves in response to 2 mM ATP, CTP, Ap_4_A, and Cp_4_C. Our experiments revealed that the NBT staining of leaves, indicating ^•^O_2_^−^ accumulation, was increased in Col-0 leaves treated with CTP, Ap_4_A, and Cp_4_C but not by eATP, while in the *dorn1-3* mutant, only Cp_4_C evoked an accumulation of ^•^O_2_^−^ (Figure 3a). DAB staining representing the concentration of H_2_O_2_ in leaves was increased in Col-0 leaves under eATP, Ap_4_A, and Cp_4_C, while CTP caused only slight DAB staining. In the *dorn1-3* mutant, only CTP and Cp_4_C evoked an accumulation of H_2_O_2_ in the leaves. Nevertheless, only weak DAB staining was caused by CTP (Figure 3b).

### 2.4. ROS Are Involved in Signal Transduction Evoked by eATP, Ap_4_A and Cp_4_C, Leading to Stomatal Closure

**Figure 4:**
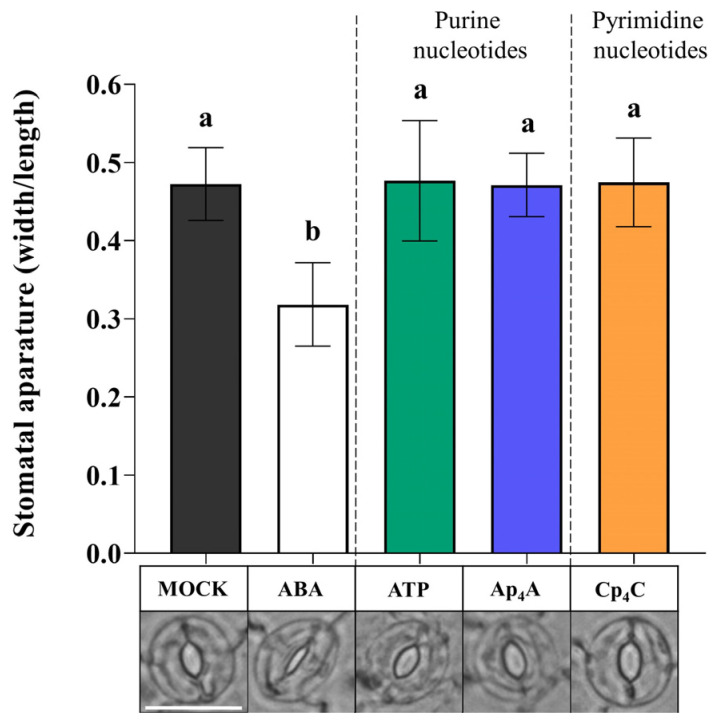
Reactive oxygen species (ROS) enzyme scavengers, catalase (CAT) and superoxide dismutase (SOD), eliminate the effect of stomatal closure after the 2 mM adenosine triphosphate (ATP), diadenosine tetraphosphate (Ap_4_A), dicytidine tetraphosphate (Cp_4_C) treatment in *Arabidopsis thaliana* Col-0 leaves. White bar = 25 μm. Bars represent mean values ± SD, *n* ≥ 20, three biological replicates. Different letters above the error bars indicate statistically significant differences according to the ANOVA analysis with the post-hoc Tukey’s HSD multiple comparisons test (*p* < 0.05).

Based on the results indicating that Ap_4_A and Cp_4_C induced the production of ROS (Figure 3a,b), we wondered whether these key signalling molecules are components of signal transduction pathways evoked by Np_n_Ns leading to stomatal closure. We simultaneously applied superoxide dismutase (SOD) and catalase (CAT), enzymes scavenging ROS [54,55], and thereby sought to confirm the role of ^•^O_2_^−^ and H_2_O_2_ in the transduction pathway of the signal generated by Ap_4_A and Cp_4_C. Interestingly, CAT and SOD eliminated the effect of stomatal closure under simultaneous nucleotide treatment, so our observations showed the direct involvement of ^•^O_2_^−^ and H_2_O_2_ in stomatal closure evoked by eATP, Ap_4_A, and Cp_4_C. However, the plants did close their stomata upon adding ABA (Figure 4).

### 2.5. P2K1/DORN1 Is Implicated in Ap_4_A- and eATP-Responsive Gene Expression

**Figure 5:**
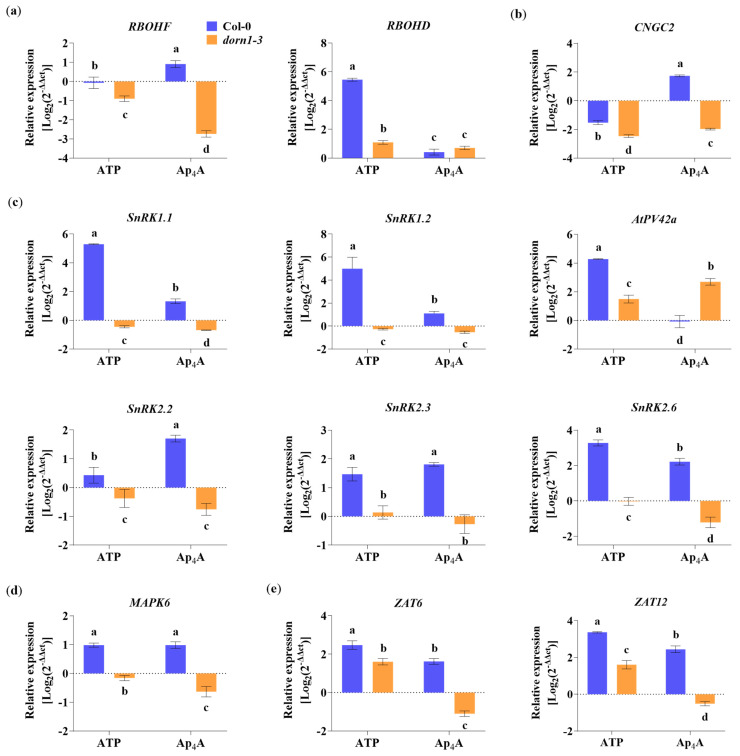
The purinoceptor P2K1/DORN1 is involved in the Ap_4_A-induced transcriptional response in *Arabidopsis thaliana* Col-0 leaves. Graphs present the changes in the gene expression level for NADPH oxidase respiratory burst homologs (*RBOHD* and *RBOHF*) (**a**), cyclic nucleotide-gated channel 2 (*CNGC2*) (**b**), SNF1/AMPK-related protein kinases (*SnRKs*) (**c**), mitogen-activated protein kinase 6 (*MAPK6*) (**d**), and transcription factors (*ZAT6* and *ZAT12*) (**e**). Leaves taken from Col-0 and the *dorn1-3* mutant were treated for 2 h with 2 mM adenosine triphosphate (ATP) and diadenosine tetraphosphate (Ap_4_A). Transcript levels are represented as Log_2_(2^–ΔΔCt^) compared to the MOCK-treated (control) plants. The housekeeping gene *AtACT2* was used for data normalisation as an endogenous control. Data are mean ± SD from 3 biological replicates. Different letters above the error bars indicate statistically significant differences according to the ANOVA analysis with the post-hoc Tukey’s HSD multiple comparisons test (*p* < 0.05).

It is known that transcriptional upregulation of defence-related and wound-response genes by eATP is P2K1/DORN1-dependent [26,56]. Thus, we decided to investigate whether Ap_4_A also changes the expression of the defence-related genes and whether the plasma membrane receptor P2K1/DORN1 is engaged in this regulation. To understand the signal transduction pathway evoked by Ap_4_A, we tested the gene expression coding for proteins as a component of signalling hubs known as key points in response to stresses. First, we studied the NADPH oxidase respiratory burst oxidase homologs (RBOHs), RBOHD, and RBOHF, which generate ROS [54]. We found that Ap_4_A up-regulated *RBOHF* but not by eATP in Col-0 plants. Interestingly, both eATP and Ap_4_A downregulated *RBOHF* expression in the *dorn1-3* mutant (Figure 5a). The expression of *RBOHD* was drastically induced (the most among all studied genes) by eATP but only in Col-0 plants. In contrast, in the *dorn1-3* plants, this effect was weak. Ap_4_A evoked slight changes in expression levels of *RBOHD* in Col-0 and *dorn1-3* plants (Figure 5a).

Other components involved in a variety of signalling pathways, ranging from development to stress responses, are cyclic nucleotide-gated channels (CNGCs) [57,58]. Moreover, AtCNGC2 mediates eATP signal transduction in cells of the root epidermis [20]. We found that Ap_4_A induced *CNGC2* expression in Col-0 plants and decreased the expression in the *dorn1-3* mutant. Extracellular ATP decreased the expression of *CNGC2* in both Col-0 and *dorn1-3* mutant plants (Figure 5b). We also focused on essential protein kinases, such as SnRKs, that regulate cellular energy homeostasis, stress response, and growth [59]. Thus, we checked the changes in the expression of *SnRK1.1*, *SnRK1.2*, *SnRK2.1*, *SnRK2.2*, and *SnRK2.6*. We also tested the expression of *PV42a* encoding cystathionine-β-synthase (CBS) domain-containing protein belonging to the PV42 class of γ-type subunits of the plant SnRK1 complexes. It is known that CBS domains generally act as regulatory domains of protein activity through adenosyl ligand binding [60]. Our experiments showed that eATP strongly induced the expression of *SnRK1.1*, *SnRK1.2*, and *PV42a* in Col-0 plants. Although Ap_4_A causes a lower effect than eATP, the elevation in the expression of *SnRK1.1* and *SnRK1.2* was statistically significant. Interestingly, in Col-0 plants, only eATP up-regulates the transcription of *PV42a*. Still, in the *dorn1-3* mutant compared to Col-0, only Ap_4_A treatment caused induction of the expression (Figure 5c). Extracellular ATP and Ap_4_A increased the expression of *SnRK2.2*, *SnRK2.3*, and *SnRK2.6* in Col-0 plants. In the *dorn1-3* mutant plants, Ap_4_A down-regulated *SnRK2.2*, *SnRK2.3*, and *SnRK2.6*. Still, the effect of eATP in the mutant was not the same for the expression of the three *SnRK2* genes; namely, the expression of *SnRK2.2* was decreased, *SnRK2.3* was slightly increased, and there was no effect on *SnRK2.6* expression (Figure 5c). The strong relationships between secondary messengers, such as ROS and MAPKs, are often highlighted in the literature [61,62]. MAPK6, among its roles in various metabolic processes in plants, can regulate the activities of diverse targets, including transcription factors [63]. We observed an up-regulation of *MAPK6* expression by both eATP and Ap_4_A in Col-0 plants and down-regulation in the *dorn1-3* mutant (Figure 5d). Among the transcription factors that MAPKs regulate, we tested the regulation of expression of the zinc-finger transcription factors (*ZAT6* and *ZAT12*) and found that eATP and Ap_4_A up-regulated the expression of both genes, as mentioned above, in Col-0 plants. Extracellular ATP increased the expression of *ZAT6* and *ZAT12* also in the *dorn1-3* mutant, but Ap_4_A downregulated the expression of both genes in the mutant plants (Figure 5e).

## 3. Discussion

Plants are exposed to continuous changes in environmental conditions that lead to an imbalance in cellular homeostasis. It is known that in response to various stresses in prokaryotic and eukaryotic cells, Np_n_Ns accumulate. The accumulation of such uncommon nucleotides can be considered in the context of the “friend hypothesis” (alarmone) and “foe hypothesis” regarding critically damaged cells as a result of internal and external stresses [33,64]. Although there are identified Ap_4_A-binding protein targets in cells [33], the signalling pathways are still unclear. We reported previously that extracellular Np_n_Ns regulate the phenylpropanoid pathway, producing secondary metabolites—key molecules in response to abiotic stress in *Arabidopsis thaliana* and *Vitis vinifera* [28,29,30]. Notably, one of the phenylpropanoid pathway enzymes, 4-coumarate:CoA ligase, is known to catalyse the synthesis of Ap_4_A [65], and its activity was increased by Ap_4_A [28]. It is known that some extracellular Ap_n_N may become internalised and operate intracellularly [33]. Despite this obvious evidence of the signalling function of uncommon nucleotides in regulating phenylpropanoid synthesis, no receptors or signalling pathways have been identified in plants until now. Here, we demonstrated, for the first time, that Ap_4_A and Cp_4_C evoked stomatal closure in *Arabidopsis thaliana* leaves (Figure 1). We did not observe such an effect in *dorn1-3* plants under the Ap_4_A effect (Figure 2). Thus, we can conclude that plasma membrane purinoreceptor P2K1/DORN1 is essential in Ap_4_A perception. However, our research also indicates that P2K1/DORN1 is not involved in signal perception elicited by Cp_4_C (Figure 2). Such results suggest that in plants, P2K1/DORN1 is not Cp_4_C-binding, or there are other protein(s) interacting with this nucleotide. After Ap_4_A signal recognition, P2K1/DORN1 stimulates ROS burst and the defence-related response. Our data indicating ROS involvement in the plant response to Ap_4_A and Cp_4_C support the hypothesis concerning the signalling function of Np_4_Ns (Figure 3 and Figure 4). Moreover, the HPLC assay proved that Ap_4_A was not degraded to corresponding mononucleotides, which could evoke stomatal closure during the experiment (Figure S1). Only a tiny amount of CTP was detected in a solution of Cp_4_C after the investigation. Still, as we proved, CTP did not evoke stomatal closure (Figure 1). Therefore, it confirms that the observed stomatal closure and ROS accumulation were caused by Ap_4_A and Cp_4_C but not by their decomposition products.

The upregulation of defence-related genes encoding proteins involved in signalling hubs was reported [59]. The expression of the genes described in this research was mostly abolished or down-regulated in the *dorn1-3* mutant (Figure 5). Recent studies consider cross-talk between diverse plant defence response markers such as ROS, hormones, and kinase cascades, leading to transcriptional, translational, and metabolic reprogramming [54]. Our transcriptional analysis focused on elements that integrate various signals and included cyclic nucleotide-gated channels (CNGCs) and NADPH oxidases—respiratory burst oxidase homologs (RBOHD and RBOHF) that generate ROS. Moreover, our studies are focused on SNF1-related protein kinases (SnRKs) and PV42a, a cystathionine-β-synthase (CBS) domain-containing protein, belongs to the PV42 class of γ-type subunits of the plant SnRK1 complexes. The next elements of signal transduction pathways that we tested concern MAPK6 and transcription factors (ZATs) (Figure 5). The transcript level of *CGNC2* increased only under Ap_4_A in Col-0 plant leaves (Figure 5b). Involving CGNC2 in another purine nucleotide, eATP, signal transduction in the root epidermis and eATP-induced Ca^2+^ influx were described by Wang [20]. This result suggests that CNGC channels can be a part of signal transduction evoked by Ap_4_A.

Rapid systemic signalling in response to stress can be stimulated by RBOHD and RBOHF, producing apoplastic ROS [66]. It is known that the elevated production of ROS and stomatal closure are mediated by eATP recognition by the receptor P2K1/DORN1, followed by direct phosphorylation of RBOHD [12], while *RBOHD* expression was significantly reduced in *dorn1-3* mutant plants. Our studies showed that transcriptomic changes in both *RBOHD* and *RBOHF* evoked by Ap_4_A are similar, but in the *dorn1-3* plants, the expression of *RBOHF* was also strongly inhibited (Figure 5a). This observation correlated with the accumulation of ROS in *Arabidopsis thaliana* leaves (Figure 3). Stress signalling in plants also involves different families of kinases, including the MAPK module, that can be activated by ROS [67]. Moreover, it was previously shown that MAPKs are activated by eATP [26,68,69,70]. We observed the induction of *MAPK6* expression evoked by eATP and Ap_4_A (Figure 5d), and it is known that MPK6 modulates actin remodelling to activate stomatal defence in *Arabidopsis thaliana* [71]. MAPK pathways are necessary for several ABA responses in many plant species, including antioxidant defence and guard cell signalling [47,48]. Protein complexes SNF1-related protein kinase 1s (SnRK1s) and SnRK2s play a prominent role in ABA signalling [72,73]. Numerous studies indicate SnRK1s and SnRK2s as regulators of the target of rapamycin (TOR) kinase activity in controlling autophagy [74,75]. We observed that Ap_4_A induced the expression of both *SnRK1*s and *SnRK2*s at a similar level in Col-0 plants. However, induction evoked by eATP was much higher for *SnRK1*s than *SnRK2*s in wild-type plants. In the *dorn1-3* mutant, the expression of *SnRK1*s and *SnRK2*s was decreased (Figure 5c). Also, neither of the tested pyrimidine nucleotides, CTP and Cp_4_C, affected the expression of *SnRK*s in Col-0 plants (Figure S2). It is known that SnRKs can regulate RBOH, which is engaged in ROS production [54]. The SnRK1s and SnRK2s were identified as critical nodes for stress and growth signalling pathways [59]. Moreover, it was suggested that under normal conditions, cytosol-localised SnRK1.1, in response to high-ammonium or low-pH stress, migrates to the nucleus and promotes the phosphorylation of the transcription factors regulating the expression of responsive genes [76]. Studies on AKINβ1, subunit SnRK1, showed its regulatory effect on secondary metabolic processes (e.g., flavonoid metabolism) [77]. Another SnRK1 subunit is PV42a, which is the CBS domain protein. Ap_4_A did not change the expression of the gene encoding AtPV42a in Col-0 plants (Figure 5c). It is known that enzymes containing CBS domains can be regulated by Ap_4_A binding [33]. Therefore, we postulate that AtPV42a regulates SnRK1s in response to Ap_4_A. Moreover, SnRK1, SnRK2, and MAPK interact with transcriptional factors [78,79]. The induction of ZAT12 and ZAT6 transcription factors in which MAPK6 is involved in an abiotic stress marker was described [63]. In the present research, we found that Ap_4_A and eATP induced both *ZAT6* and *ZAT12* gene expression in Col-0 plants, and lack of the P2K1/DORN1 receptor in the *dorn1-3* mutants diminished this effect (Figure 5e). It is known that the transcript level of *ZAT6* positively affected the concentrations of phenylpropanoids, including anthocyanin and total flavonoids [80]. Moreover, it was proved that ZAT6 and ZAT12 are involved in the response to cadmium stress and abiotic stress in plants [81,82,83,84], and the expression of *ZAT12* was strictly dependent on the ROS wave [85,86].

**Figure 6:**
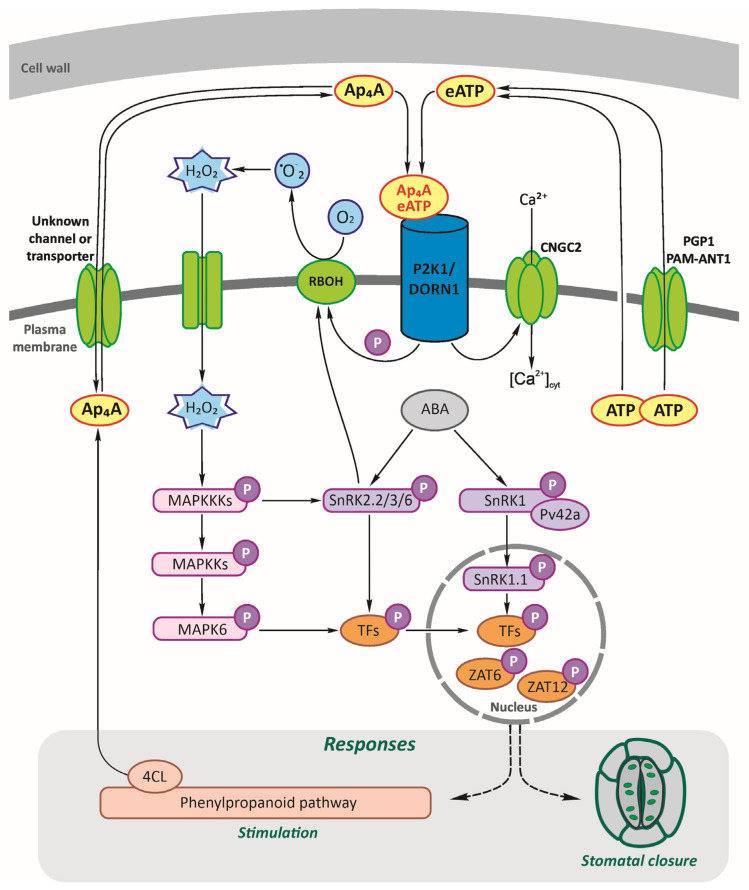
Hypothetical working model of diadenosine tetraphosphate (Ap_4_A) signalling network in a plant cell. Ap_4_A, similar to extracellular adenosine triphosphate (eATP) [26], can be recognised by the purinoreceptor P2K1/DORN1 and lead to stomatal closure. As our study showed, Ap_4_A triggered the reactive oxygen species (ROS) wave, which evoked changes in the expression of the defence-related genes encoding proteins involved in signalling hubs, such as CNGC2; RBOHD and RBOHF generate ROS; SnRKs; AtPV42a, γ-type subunits of the plant SnRK1 complexes; MAPK cascades; and transcription factors, ZATs. The wounded cell membrane and transporters can release ATP to the extracellular space matrix: PGP1, p-glycoprotein belonging to ATP-binding cassette ABC transporters, and PM-ANT1, plasma membrane-localised nucleotide transporters [15,87]. Extracellular ATP recognition by P2K1/DORN1 evoked phosphorylation of RBOHD [12]. Also, CNGC2 [20] and MAPK cascades are involved in eATP signal transduction [26,68,69,70]. We previously described that 4-coumarate:CoA ligase (4CL), the branch point of the phenylpropanoid pathway, can synthesise Ap_4_A [65], and its activity is induced by Ap_4_A [28]. As yet, no channel or transporter for Ap_4_A in plants is known. P, phosphate.

The results of our research presented here shed more light on the signalling function of Ap_4_A, its perception and signal transduction pathway in plants. We had previously proposed a hypothetical Np_n_N signalling network in a plant cell [2]. Then, we strongly suggested the existence of some receptor and signalling transduction pathways involving signalling hubs and transcription factors resulting in gene expression changes, including genes coding for enzymes catalysing the phenylpropanoid pathway [2,28,29,30]. Here, we fill a few gaps in this network (Figure 6).

## 4. Materials and Methods

### 4.1. Nucleotides

Ap_4_A and Cp_4_C were synthesised following previously reported procedures, purified by reverse-phase HPLC, and isolated as ammonium (NH_4_^+^) salts. The purities (>95%) were confirmed by analytical HPLC, ^1^H NMR and ^31^P NMR [30].

### 4.2. Plant Material

*Arabidopsis thaliana* lines were in the Columbia (Col-0) ecotype. A T-DNA insertion line of LecRK-I.9 (Salk_042209; *dorn1-3*) was obtained from NASC (Nottingham Arabidopsis Stock Centre, Nottingham, UK). Surface-sterilised seeds were stratified in darkness at 4 °C for 48 h and transferred to a growth chamber. Plants were grown for four weeks on the soil at 21–23 °C, 60–70% humidity, under a long-day photoperiod (16 h light and 8 h dark), 120 µmol m^−2^ s^−1^ light intensity. Genotyping of insertional mutants is described in Methods S1. Primers are listed in Table S1.

### 4.3. Stomatal Aperture Measurement

To ensure fully open stomata, plants were placed for 3 h under light intensity 120 µmol m^−2^ s^−1^. Samples of leaf epidermis were obtained from the abaxial side. They were placed on a microscope slide for 2 h of incubation in (i) MOCK solution MES/KOH opening buffer containing 10 mM MES pH 6.15, 10 mM KCl, 10 μM CaCl_2_ (control), (ii) 10 µM abscisic acid (ABA, Sigma Aldrich, St. Louis, MO, USA, A1049) dissolved in the MOCK solution MES/KOH buffer, and (iii) 2 mM ADP (Sigma, A2754), ATP (Sigma, AA8937), Ap_3_A, Ap_4_A, and CDP (Sigma, C9755), CTP, Cp_3_C, Cp_4_C dissolved in the MOCK solution MES/KOH buffer. We chose 2 mM concentration of nucleotides based on literature data concerning the effect of eATP on regulation of stomatal aperture [12].

CTP and Np_n_Ns were synthesised as described previously [30]. Stomata were observed using the ZOE Fluorescent Cell Imager (Bio-Rad, Hercules, CA, USA, 1450031EDU). Measurements, including stomatal aperture width and length, were performed with ImageJ 1.54g software. The involvement of ROS in stomatal movement under nucleotide treatment was examined by the simultaneous addition of ROS enzyme scavengers to the nucleotide solutions. Catalase (CAT) (Sigma Aldrich, C100) and superoxide dismutase (SOD) (Sigma Aldrich, S9697), in a concentration of 100 units mL^−1^ and 500 units mL^−1^, respectively, were used together in an incubation mixture.

### 4.4. Detection of Intracellular ROS Burst in Leaves

Two leaves were incubated in 3 mL of MOCK solution MES/KOH opening buffer or the buffer enriched in 2 mM concentrations of tested nucleotides. After 2 h, the incubating buffers were gently replaced with 3 mL of staining solutions, and submerged leaves were vacuum infiltrated three times (1 min each time). The staining solution for ^•^O_2_^−^ detection was composed of 0.5% nitroblue tetrazolium (NBT, Sigma-Aldrich, N6876) dissolved in 10 mM potassium phosphate buffer, pH 7.8 [88], and the staining solution for H_2_O_2_ synthesis was composed of 3,3′-diaminobenzidine tetrahydrochloride (DAB, Sigma-Aldrich, D5905) (1 mg ml^−1^ DAB) dissolved in 10 mM potassium phosphate buffer, pH 7.4, and 0.05% Tween [89]. Samples were incubated at room temperature for the next 2 h in the dark with continuous shaking. Then, leaves were incubated in 96% ethanol overnight for bleaching, and the photographs were taken with an Epson Perfection V700 scanner.

### 4.5. Gene Expression Analyses

According to the manufacturer’s instructions, total RNA was extracted from leaves using the RNeasy Plant Mini Kit (Qiagen, Germantown MD, USA). Evaluation of RNA purity, cDNA synthesis, reverse transcription, and RT-qPCR were performed as described previously by Pietrowska-Borek and co-workers [28,90,91]. The qRT-PCR reactions were performed using a CFX96 Real-Time PCR Detection System (Bio-Rad). The specific primers for *Arabidopsis thaliana* genes are listed in Table S1. The 2^−ΔΔCt^ method [92] was applied to calculate the relative gene expression. The data were normalised against the reference gene, *ACTIN2* (*ACT2*). For statistical analysis, the gene expression data were Log_2_-transformed to meet distribution and variance assumptions.

### 4.6. Statistical Analysis

All experiments were performed at least three times. The results are shown as the mean ± SD. The statistical significance of the differences among the means was analysed by the ANOVA with the post-hoc Tukey’s HSD multiple comparisons test (*p* < 0.05) using Statistica, Version 13 (TIBCO Software Inc., Palo Alto, CA, USA).

## 5. Conclusions

In the present work, we confirmed that in plants, an Ap_4_A receptor exists, and we found that it is purine receptor P2K1/DORN1. Moreover, we indicated ROSs as second messengers, kinases, and transcription factors engaged in the Ap_4_A signal transduction pathway. Nevertheless, further studies, both in silico and in vitro, on the binding of Ap4A to the P2K1/DORN1, including key residues that modulate Ap4A affinity, are required. We believe that the presented results in this paper contribute to the description of the role of Np_n_Ns in signalling hubs and can help better understand the function of uncommon nucleotides in plants.

## Data Availability

The original data obtained during this research are available from the corresponding authors on reasonable request, and some of them are also accessible in the Supplementary materials.

## Supplementary Materials

The supporting information can be downloaded at: https://www.mdpi.com/article/10.3390/ijms242316688/s1. Refs. [93,94,95,96,97,98,99,100] are cited in the supplementary materials.

## References

[1] Sun T., Zhang Y. (2021). Short- and Long-distance Signaling in Plant Defense. Plant J..

[2] Pietrowska-Borek M., Dobrogojski J., Sobieszczuk-Nowicka E., Borek S. (2020). New Insight into Plant Signaling: Extracellular ATP and Uncommon Nucleotides. Cells.

[3] Kim S.Y., Sivaguru M., Stacey G. (2006). Extracellular ATP in Plants. Visualization, Localization, and Analysis of Physiological Significance in Growth and Signaling. Plant Physiol..

[4] Wu J., Steinebrunner I., Sun Y., Butterfield T., Torres J., Arnold D., Gonzalez A., Jacob F., Reichler S., Roux S.J. (2007). Apyrases (Nucleoside Triphosphate-Diphosphohydrolases) Play a Key Role in Growth Control in *Arabidopsis*. Plant Physiol..

[5] Riewe D., Grosman L., Fernie A.R., Wucke C., Geigenberger P. (2008). The Potato-Specific Apyrase Is Apoplastically Localized and Has Influence on Gene Expression, Growth, and Development. Plant Physiol..

[6] Tonón C., Cecilia Terrile M., José Iglesias M., Lamattina L., Casalongué C. (2010). Extracellular ATP, Nitric Oxide and Superoxide Act Coordinately to Regulate Hypocotyl Growth in Etiolated *Arabidopsis* Seedlings. J. Plant Physiol..

[7] Clark G., Wu M., Wat N., Onyirimba J., Pham T., Herz N., Ogoti J., Gomez D., Canales A.A., Aranda G. (2010). Both the Stimulation and Inhibition of Root Hair Growth Induced by Extracellular Nucleotides in *Arabidopsis* Are Mediated by Nitric Oxide and Reactive Oxygen Species. Plant Mol. Biol..

[8] Zhu R., Dong X., Xue Y., Xu J., Zhang A., Feng M., Zhao Q., Xia S., Yin Y., He S. (2020). Redox-Responsive Transcription Factor 1 (RRFT1) Is Involved in Extracellular ATP-Regulated *Arabidopsis thaliana* Seedling Growth. Plant Cell Physiol..

[9] Reichler S.A., Torres J., Rivera A.L., Cintolesi V.A., Clark G., Roux S.J. (2009). Intersection of Two Signalling Pathways: Extracellular Nucleotides Regulate Pollen Germination and Pollen Tube Growth via Nitric Oxide. J. Exp. Bot..

[10] Wu Y., Qin B., Feng K., Yan R., Kang E., Liu T., Shang Z. (2018). Extracellular ATP Promoted Pollen Germination and Tube Growth of *Nicotiana Tabacum* through Promoting K^+^ and Ca^2+^ Absorption. Plant Reprod..

[11] Chivasa S., Ndimba B.K., Simon W.J., Lindsey K., Slabas A.R. (2005). Extracellular ATP Functions as an Endogenous External Metabolite Regulating Plant Cell Viability. Plant Cell.

[12] Chen D., Cao Y., Li H., Kim D., Ahsan N., Thelen J., Stacey G. (2017). Extracellular ATP Elicits DORN1-Mediated RBOHD Phosphorylation to Regulate Stomatal Aperture. Nat. Commun..

[13] Tripathi D., Zhang T., Koo A.J., Stacey G., Tanaka K. (2018). Extracellular ATP Acts on Jasmonate Signaling to Reinforce Plant Defense. Plant Physiol..

[14] Goodman H.L., Kroon J.T.M., Tomé D.F.A., Hamilton J.M.U., Alqarni A.O., Chivasa S. (2022). Extracellular ATP Targets *Arabidopsis* RIBONUCLEASE 1 to Suppress Mycotoxin Stress-Induced Cell Death. New Phytol..

[15] Thomas C., Rajagopal A., Windsor B., Dudler R., Lloyd A., Roux S.J. (2000). A Role for Ectophosphatase in Xenobiotic Resistance. Plant Cell.

[16] Kim S.H., Yang S.H., Kim T.J., Han J.S., Suh J.W. (2009). Hypertonic Stress Increased Extracellular ATP Levels and the Expression of Stress-Responsive Genes in *Arabidopsis thaliana* Seedlings. Biosci. Biotechnol. Biochem..

[17] Sun J., Zhang X., Deng S., Zhang C., Wang M., Ding M., Zhao R., Shen X., Zhou X., Lu C. (2012). Extracellular ATP Signaling Is Mediated by H_2_O_2_ and Cytosolic Ca^2+^ in the Salt Response of *Populus euphratica* Cells. PLoS ONE.

[18] Hou Q.Z., Sun K., Zhang H., Su X., Fan B.Q., Feng H.Q. (2018). The Responses of Photosystem II and Intracellular ATP Production of *Arabidopsis* Leaves to Salt Stress Are Affected by Extracellular ATP. J. Plant Res..

[19] Duong H.N., Cho S.H., Wang L., Pham A.Q., Davies J.M., Stacey G. (2021). Cyclic Nucleotide-Gated Ion Channel 6 Is Involved in Extracellular ATP Signaling and Plant Immunity. Plant J..

[20] Wang L., Ning Y., Sun J., Wilkins K.A., Matthus E., McNelly R.E., Dark A., Rubio L., Moeder W., Yoshioka K. (2022). *Arabidopsis thaliana* CYCLIC NUCLEOTIDE-GATED CHANNEL2 Mediates Extracellular ATP Signal Transduction in Root Epidermis. New Phytol..

[21] Foresi N.P., Laxalt A.M., Tonón C.V., Casalongué C.A., Lamattina L. (2007). Extracellular ATP Induces Nitric Oxide Production in Tomato Cell Suspensions. Plant Physiol..

[22] Wu S.-J., Wu J.-Y. (2008). Extracellular ATP-Induced NO Production and Its Dependence on Membrane Ca^2+^ Flux in *Salvia miltiorrhiza* Hairy Roots. J. Exp. Bot..

[23] Song C.J., Steinebrunner I., Wang X., Stout S.C., Roux S.J. (2006). Extracellular ATP Induces the Accumulation of Superoxide via NADPH Oxidases in *Arabidopsis*. Plant Physiol..

[24] Wu S.-J., Liu Y.-S., Wu J.-Y. (2008). The Signaling Role of Extracellular ATP and Its Dependence on Ca^2+^ Flux in Elicitation of *Salvia miltiorrhiza* Hairy Root Cultures. Plant Cell Physiol..

[25] Demidchik V., Shang Z., Shin R., Thompson E., Rubio L., Laohavisit A., Mortimer J.C., Chivasa S., Slabas A.R., Glover B.J. (2009). Plant Extracellular ATP Signalling by Plasma Membrane NADPH Oxidase and Ca^2+^ Channels. Plant J..

[26] Choi J., Tanaka K., Cao Y., Qi Y., Qiu J., Liang Y., Lee S.Y., Stacey G. (2014). Identification of a Plant Receptor for Extracellular ATP. Science.

[27] Li P., Zhao L., Qi F., Htwe N.M.P.S., Li Q., Zhang D., Lin F., Shang-Guan K., Liang Y. (2021). The Receptor-like Cytoplasmic Kinase RIPK Regulates Broad-Spectrum ROS Signaling in Multiple Layers of Plant Immune System. Mol. Plant.

[28] Pietrowska-Borek M., Nuc K., Zielezińska M., Guranowski A. (2011). Diadenosine Polyphosphates (Ap_3_A and Ap_4_A) Behave as Alarmones Triggering the Synthesis of Enzymes of the Phenylpropanoid Pathway in *Arabidopsis thaliana*. FEBS Open Bio.

[29] Pietrowska-Borek M., Czekała Ł., Belchí-Navarro S., Pedreño M.A., Guranowski A. (2014). Diadenosine Triphosphate Is a Novel Factor Which in Combination with Cyclodextrins Synergistically Enhances the Biosynthesis of *Trans*-Resveratrol in *Vitis vinifera* Cv. Monastrell Suspension Cultured Cells. Plant Physiol. Biochem..

[30] Pietrowska-Borek M., Wojdyła-Mamoń A., Dobrogojski J., Młynarska-Cieślak A., Baranowski M.R., Dąbrowski J.M., Kowalska J., Jemielity J., Borek S., Pedreño M.A. (2020). Purine and Pyrimidine Dinucleoside Polyphosphates Differentially Affect the Phenylpropanoid Pathway in *Vitis vinifera* L. Cv. Monastrell Suspension Cultured Cells. Plant Physiol. Biochem..

[31] Dixon R.A., Paiva N.L. (1995). Stress-Induced Phenylpropanoid Metabolism. Plant Cell.

[32] Sharma A., Shahzad B., Rehman A., Bhardwaj R., Landi M., Zheng B. (2019). Response of Phenylpropanoid Pathway and the Role of Polyphenols in Plants under Abiotic Stress. Molecules.

[33] Ferguson F., McLennan A.G., Urbaniak M.D., Jones N.J., Copeland N.A. (2020). Re-Evaluation of Diadenosine Tetraphosphate (Ap_4_A) from a Stress Metabolite to Bona Fide Secondary Messenger. Front. Mol. Biosci..

[34] Lee P.C., Bochner B.R., Ames B.N. (1983). AppppA, Heat-Shock Stress, and Cell Oxidation. Proc. Natl. Acad. Sci. USA.

[35] Bochner B.R., Lee P.C., Wilson S.W., Cutler C.W., Ames B.N. (1984). AppppA and Related Adenylylated Nucleotides Are Synthesized as a Consequence of Oxidation Stress. Cell.

[36] Baltzinger M., Ebel J.-P., Remy P. (1986). Accumulation of Dinucleoside Polyphosphates in *Saccharomyces cerevisiae* under Stress Conditions. High Levels Are Associated with Cell Death. Biochimie.

[37] Coste H., Brevet A., Plateau P., Blanquet S. (1987). Non-Adenylylated Bis(5’-Nucleosidyl) Tetraphosphates Occur in *Saccharomyces cerevisiae* and in *Escherichia coli* and Accumulate upon Temperature Shift or Exposure to Cadmium. J. Biol. Chem..

[38] Pálfi Z., Surányi G., Borbély G. (1991). Alterations in the Accumulation of Adenylylated Nucleotides in Heavy-Metal-Ion-Stressed and Heat-Stressed *Synechococcus* Sp. Strain PCC 6301, a Cyanobacterium, in Light and Dark. Biochem. J..

[39] Pham A.Q., Cho S.-H., Nguyen C.T., Stacey G. (2020). *Arabidopsis* Lectin Receptor Kinase P2K2 Is a Second Plant Receptor for Extracellular ATP and Contributes to Innate Immunity. Plant Physiol..

[40] Cho S.-H., Nguyen C.T., Pham A.Q., Stacey G. (2023). Computational Prediction and *in Vitro* Analysis of the Potential Ligand Binding Site within the Extracellular ATP Receptor, P2K2. Plant Signal. Behav..

[41] Jose J., Ghantasala S., Choudhury S.R. (2020). *Arabidopsis* Transmembrane Receptor-like Kinases (RLKS): A Bridge between Extracellular Signal and Intracellular Regulatory Machinery. Int. J. Mol. Sci..

[42] Vigne P., Breittmayer J.P., Frelin C. (2000). Diadenosine Polyphosphates as Antagonists of the Endogenous P2Y_1_ Receptor in Rat Brain Capillary Endothelial Cells of the B7 and B10 Clones: AP_n_As as Antagonists of P2Y_1_ Receptors. Br. J. Pharmacol..

[43] McDonald H.A., Chu K.L., Bianchi B.R., McKenna D.G., Briggs C.A., Burgard E.C., Lynch K.J., Faltynek C., Cartmell J., Jarvis M.F. (2002). Potent Desensitization of Human P2X3 Receptors by Diadenosine Polyphosphates. Eur. J. Pharmacol..

[44] Wang Y., Chang C.-F., Morales M., Chiang Y.-H., Harvey B.K., Su T.-P., Tsao L.-I., Chen S., Thiemermann C. (2003). Diadenosine Tetraphosphate Protects against Injuries Induced by Ischemia and 6-Hydroxydopamine in Rat Brain. J. Neurosci..

[45] Verspohl E.J., Johannwille B., Kaiserling-Buddemeier I., Schlüter H., Hagemann J. (2010). Diadenosine Polyphosphates in Cultured Vascular Smooth-Muscle Cells and Endothelium Cells—Their Interaction with Specific Receptors and Their Degradation. J. Pharm. Pharmacol..

[46] Burnstock G. (2018). Purine and Purinergic Receptors. Brain Neurosci. Adv..

[47] Hsu P.-K., Dubeaux G., Takahashi Y., Schroeder J.I. (2021). Signaling Mechanisms in Abscisic Acid-Mediated Stomatal Closure. Plant J. Cell Mol. Biol..

[48] Danquah A., De Zelicourt A., Colcombet J., Hirt H. (2014). The Role of ABA and MAPK Signaling Pathways in Plant Abiotic Stress Responses. Biotechnol. Adv..

[49] Bharath P., Gahir S., Raghavendra A.S. (2021). Abscisic Acid-Induced Stomatal Closure: An Important Component of Plant Defense against Abiotic and Biotic Stress. Front. Plant Sci..

[50] Guranowski A. (2004). Metabolism of Diadenosine Tetraphosphate (Ap_4_A) and Related Nucleotides in Plants; Review with Historical and General Perspective. Front. Biosci..

[51] Waszczak C., Carmody M., Kangasjarvi J. (2018). Reactive Oxygen Species in Plant Signaling. Annu. Rev. Plant Biol..

[52] Smirnoff N., Arnaud D. (2019). Hydrogen Peroxide Metabolism and Functions in Plants. New Phytol..

[53] Macho A.P., Zipfel C. (2014). Plant PRRs and the Activation of Innate Immune Signaling. Mol. Cell.

[54] Mittler R., Zandalinas S.I., Fichman Y., Van Breusegem F. (2022). Reactive Oxygen Species Signalling in Plant Stress Responses. Nat. Rev. Mol. Cell Biol..

[55] Khokon A.R., Okuma E., Hossain M.A., Munemasa S., Uraji M., Nakamura Y., Mori I.C., Murata Y. (2011). Involvement of Extracellular Oxidative Burst in Salicylic Acid-Induced Stomatal Closure in *Arabidopsis*: Extracellular ROS Mediate SA-Induced Stomatal Closure. Plant Cell Environ..

[56] Jewell J.B., Sowders J.M., He R., Willis M.A., Gang D.R., Tanaka K. (2019). Extracellular ATP Shapes a Defense-Related Transcriptome Both Independently and along with Other Defense Signaling Pathways. Plant Physiol..

[57] Duszyn M., Świeżawska B., Szmidt-Jaworska A., Jaworski K. (2019). Cyclic Nucleotide Gated Channels (CNGCs) in Plant Signalling-Current Knowledge and Perspectives. J. Plant Physiol..

[58] Jarratt-Barnham E., Wang L., Ning Y., Davies J.M. (2021). The Complex Story of Plant Cyclic Nucleotide-Gated Channels. Int. J. Mol. Sci..

[59] Zhang H., Zhao Y., Zhu J.-K. (2020). Thriving under Stress: How Plants Balance Growth and the Stress Response. Dev. Cell.

[60] Baudry K., Barbut F., Domenichini S., Guillaumot D., Thy M.P., Vanacker H., Majeran W., Krieger-Liszkay A., Issakidis-Bourguet E., Lurin C. (2022). Adenylates Regulate *Arabidopsis* Plastidial Thioredoxin Activities through the Binding of a CBS Domain Protein. Plant Physiol..

[61] Matsushita M., Nakamura T., Moriizumi H., Miki H., Takekawa M. (2020). Stress-Responsive MTK1 SAPKKK Serves as a Redox Sensor That Mediates Delayed and Sustained Activation of SAPKs by Oxidative Stress. Sci. Adv..

[62] Byrne D.P., Shrestha S., Galler M., Cao M., Daly L.A., Campbell A.E., Eyers C.E., Veal E.A., Kannan N., Eyers P.A. (2020). Aurora A Regulation by Reversible Cysteine Oxidation Reveals Evolutionarily Conserved Redox Control of Ser/Thr Protein Kinase Activity. Sci. Signal..

[63] Smékalová V., Doskočilová A., Komis G., Šamaj J. (2014). Crosstalk between Secondary Messengers, Hormones and MAPK Modules during Abiotic Stress Signalling in Plants. Biotechnol. Adv..

[64] McLennan A. (2000). Dinucleoside Polyphosphates—Friend or Foe?. Pharmacol. Ther..

[65] Pietrowska-Borek M., Stuible H.-P., Kombrink E., Guranowski A. (2003). 4-Coumarate:Coenzyme A Ligase Has the Catalytic Capacity to Synthesize and Reuse Various (Di)Adenosine Polyphosphates. Plant Physiol..

[66] Choi W.-G., Hilleary R., Swanson S.J., Kim S.-H., Gilroy S. (2016). Rapid, Long-Distance Electrical and Calcium Signaling in Plants. Annu. Rev. Plant Biol..

[67] Meng X., Zhang S. (2013). MAPK Cascades in Plant Disease Resistance Signaling. Annu. Rev. Phytopathol..

[68] Medina-Castellanos E., Esquivel-Naranjo E.U., Heil M., Herrera-Estrella A. (2014). Extracellular ATP Activates MAPK and ROS Signaling during Injury Response in the Fungus *Trichoderma atroviride*. Front. Plant Sci..

[69] Chen D., Hao F., Mu H., Ahsan N., Thelen J.J., Stacey G. (2021). S-Acylation of P2K1 Mediates Extracellular ATP-Induced Immune Signaling in *Arabidopsis*. Nat. Commun..

[70] Cho S.-H., Tóth K., Kim D., Vo P.H., Lin C.-H., Handakumbura P.P., Ubach A.R., Evans S., Paša-Tolić L., Stacey G. (2022). Activation of the Plant Mevalonate Pathway by Extracellular ATP. Nat. Commun..

[71] Zou M., Guo M., Zhou Z., Wang B., Pan Q., Li J., Zhou J.-M., Li J. (2021). MPK3- and MPK6-Mediated VLN3 Phosphorylation Regulates Actin Dynamics during Stomatal Immunity in *Arabidopsis*. Nat. Commun..

[72] Jossier M., Bouly J.-P., Meimoun P., Arjmand A., Lessard P., Hawley S., Grahame Hardie D., Thomas M. (2009). SnRK1 (SNF1-Related Kinase 1) Has a Central Role in Sugar and ABA Signalling in *Arabidopsis thaliana*. Plant J..

[73] Ou X., Li T., Zhao Y., Chang Y., Wu L., Chen G., Day B., Jiang K. (2022). Calcium-Dependent ABA Signaling Functions in Stomatal Immunity by Regulating Rapid SA Responses in Guard Cells. J. Plant Physiol..

[74] Signorelli S., Tarkowski Ł.P., Van den Ende W., Bassham D.C. (2019). Linking Autophagy to Abiotic and Biotic Stress Responses. Trends Plant Sci..

[75] Belda-Palazón B., Adamo M., Valerio C., Ferreira L.J., Confraria A., Reis-Barata D., Rodrigues A., Meyer C., Rodriguez P.L., Baena-González E. (2020). A Dual Function of SnRK2 Kinases in the Regulation of SnRK1 and Plant Growth. Nat. Plants.

[76] Sun D., Fang X., Xiao C., Ma Z., Huang X., Su J., Li J., Wang J., Wang S., Luan S. (2021). Kinase SnRK1.1 Regulates Nitrate Channel SLAH3 Engaged in Nitrate-Dependent Alleviation of Ammonium Toxicity. Plant Physiol..

[77] Wang Y., Wang L., Micallef B.J., Tetlow I.J., Mullen R.T., Feil R., Lunn J.E., Emes M.J. (2020). AKINβ1, a Subunit of SnRK1, Regulates Organic Acid Metabolism and Acts as a Global Modulator of Genes Involved in Carbon, Lipid, and Nitrogen Metabolism. J. Exp. Bot..

[78] Zhang M., Zhang Q., Tian C., Liu G., Pan Y., Xu X., Shi X., Zhang Z., Meng L. (2022). Physiological and Transcriptome Analyses of CaCl_2_ Treatment to Alleviate Chilling Injury in Pineapple. Plants.

[79] Son S., Im J.H., Ko J.-H., Han K.-H. (2023). SNF1-Related Protein Kinase 1 Represses *Arabidopsis* Growth through Post-Translational Modification of E2Fa in Response to Energy Stress. New Phytol..

[80] Shi H., Liu G., Wei Y., Chan Z. (2018). The Zinc-Finger Transcription Factor ZAT6 Is Essential for Hydrogen Peroxide Induction of Anthocyanin Synthesis in *Arabidopsis*. Plant Mol. Biol..

[81] Opdenakker K., Remans T., Keunen E., Vangronsveld J., Cuypers A. (2012). Exposure of *Arabidopsis thaliana* to Cd or Cu Excess Leads to Oxidative Stress Mediated Alterations in MAPKinase Transcript Levels. Environ. Exp. Bot..

[82] Shi H., Wang X., Ye T., Chen F., Deng J., Yang P., Zhang Y., Chan Z. (2014). The Cysteine2/Histidine2-Type Transcription Factor ZINC FINGER OF ARABIDOPSIS THALIANA6 Modulates Biotic and Abiotic Stress Responses by Activating Salicylic Acid-Related Genes and C-REPEAT-BINDING FACTOR Genes in *Arabidopsis*. Plant Physiol..

[83] Chen J., Yang L., Yan X., Liu Y., Wang R., Fan T., Ren Y., Tang X., Xiao F., Liu Y. (2016). Zinc-Finger Transcription Factor ZAT6 Positively Regulates Cadmium Tolerance through the Glutathione-Dependent Pathway in *Arabidopsis*. Plant Physiol..

[84] Dang F., Li Y., Wang Y., Lin J., Du S., Liao X. (2022). ZAT10 Plays Dual Roles in Cadmium Uptake and Detoxification in *Arabidopsis*. Front. Plant Sci..

[85] Brumbarova T., Le C.T.T., Ivanov R., Bauer P. (2016). Regulation of ZAT12 Protein Stability: The Role of Hydrogen Peroxide. Plant Signal. Behav..

[86] Myers R.J., Fichman Y., Stacey G., Mittler R. (2022). Extracellular ATP Plays an Important Role in Systemic Wound Response Activation. Plant Physiol..

[87] Rieder B., Neuhaus H.E. (2011). Identification of an *Arabidopsis* Plasma Membrane-Located ATP Transporter Important for Anther Development. Plant Cell.

[88] Müller K., Carstens A.C., Linkies A., Torres M.A., Leubner-Metzger G. (2009). The NADPH-oxidase *AtrbohB* Plays a Role in *Arabidopsis* Seed After-ripening. New Phytol..

[89] Daudi A., O’Brien J.A. (2012). Detection of Hydrogen Peroxide by DAB Staining in *Arabidopsis* Leaves. Bio-Protocol.

[90] Pietrowska-Borek M., Nuc K., Guranowski A. (2015). Exogenous Adenosine 5’-Phosphoramidate Behaves as a Signal Molecule in Plants; It Augments Metabolism of Phenylpropanoids and Salicylic Acid in *Arabidopsis thaliana* Seedlings. Plant Physiol. Biochem..

[91] Pietrowska-Borek M., Nuc K. (2013). Both Cyclic-AMP and Cyclic-GMP Can Act as Regulators of the Phenylpropanoid Pathway in *Arabidopsis thaliana* Seedlings. Plant Physiol. Biochem..

[92] Schmittgen T.D., Livak K.J. (2008). Analyzing Real-Time PCR Data by the Comparative CT Method. Nat. Protoc..

[93] Doyle J.L., Doyle J.M. (1987). A Rapid DNA Isolation Procedure for Small Quantities of Fresh Leaf Tissue. Phytochem. Bull..

[94] Nakashima K., Fujita Y., Kanamori N., Katagiri T., Umezawa T., Kidokoro S., Maruyama K., Yoshida T., Ishiyama K., Kobayashi M. (2009). Three *Arabidopsis* SnRK2 Protein Kinases, SRK2D/SnRK2.2, SRK2E/SnRK2.6/OST1 and SRK2I/SnRK2.3, Involved in ABA Signaling Are Essential for the Control of Seed Development and Dormancy. Plant Cell Physiol..

[95] Fang L., Hou X., Lee L.Y.C., Liu L., Yan X., Yu H. (2011). AtPV42a and AtPV42b Redundantly Regulate Reproductive Development in *Arabidopsis thaliana*. PLoS ONE.

[96] Kannan P., Pandey D., Gupta A.K., Punetha H., Taj G., Kumar A. (2012). Expression Analysis of MAP2K9 and MAPK6 during Pathogenesis of Alternaria Blight in *Arabidopsis thaliana* Ecotype Columbia. Mol. Biol. Rep..

[97] Le C.T.T., Brumbarova T., Ivanov R., Stoof C., Weber E., Mohrbacher J., Fink-Straube C., Bauer P. (2016). ZINC FINGER OF ARABIDOPSIS THALIANA12 (ZAT12) Interacts with FER-LIKE IRON DEFICIENCY-INDUCED TRANSCRIPTION FACTOR (FIT) Linking Iron Deficiency and Oxidative Stress Responses. Plant Physiol..

[98] Morales J., Kadota Y., Zipfel C., Molina A., Torres M.-A. (2016). The Arabidopsis NADPH Oxidases *RbohD* and *RbohF* Display Differential Expression Patterns and Contributions during Plant Immunity. EXBOTJ.

[99] Guranowski A., Starzyńska E., Pietrowska-Borek M., Rejman D., Blackburn G.M. (2009). Novel Diadenosine Polyphosphate Analogs with Oxymethylene Bridges Replacing Oxygen in the Polyphosphate Chain: Potential Substrates and/or Inhibitors of Ap4A Hydrolases. FEBS J..

[100] Guranowski A., Wojdyła A.M., Pietrowska-Borek M., Bieganowski P., Khurs E.N., Cliff M.J., Blackburn G.M., Błaziak D., Stec W.J. (2008). Fhit Proteins Can Also Recognize Substrates Other than Dinucleoside Polyphosphates. FEBS Lett..

