# Supplementary material for: The Plasma Membrane Purinoreceptor P2K1/DORN1 Is Essential in Stomatal Closure Evoked by Extracellular Diadenosine Tetraphosphate (Ap4A) in Arabidopsis thaliana
Source: Int J Mol Sci. 2023 Nov 24;24(23):16688. doi: 10.3390/ijms242316688 (PMC10706190; doi:10.3390/ijms242316688)
Supplement: Supplementary file 1 [file ijms-24-16688-s001.zip › ijms-2715355-supplementary.pdf]

Supplementary Materials

The plasma membrane purinoreceptor P2K1/DORN1 is essential in stomatal closure evoked by extracellular diadenosine tetraphosphate (Ap_4A) but not by dicytidine tetraphosphate (Cp_4C) in *Arabidopsis thaliana*

Jędrzej Dobrogojski, Van Hai Nguyen, Joanna Kowalska, Sławomir Borek and Małgorzata Pietrowska-Borek

Methods S1. Genotyping *dorn1-3* insertional mutant.

A T-DNA insertion line of LecRK-I.9 (Salk_042209; *dorn1-3*) was obtained from the Nottingham Arabidopsis Stock Centre (NASC, UK). Genomic DNA was extracted from 4-week-old *Arabidopsis thaliana* leaves using a slightly modified hexadecyltrimethylammonium bromide (CTAB) protocol [1]. Homozygosity for the T-DNA insertion was confirmed by PCR-based genotyping using the specific primers listed in Table **S1**. PCR conditions were as follows: 35 cycles at 95°C for 30 s, 50°C for 15 s, and 72°C for 1:40 min. Then, gel electrophoresis (1% (w/v) agarose in 1X TBE buffer) was conducted for gene product visualisation and final homozygosity confirmation.

Table S1. List of primers used for genotyping T-DNA mutant and qPCR.

Purpose	Gene ID (TAIR)	Primers	Sequence (5'→3')	Product length	Source
**Genotype**					
p2k1-3 mutant	**AT5G60300**	*mlecat5g60300-s*	TCCATGCAACAGTTGCGTTGTCT		[2]
		P2K1_R	CTGCAATACCCAAACAGTGGTA		
		LBb1	GCGTGGACCGCTTGCTGCAACT		
**qPCR**					
	**AT3G01090**	*SnRK1.1_F*	CCGCTCCAGAGGTAATTTCG	68 bp	[3]
		SnRK1.1_R	CACACCACAGCTCCAGACATCT		
	**AT3G29160**	*SnRK1.2_F*	CACCATTCCTGAGATCCGTCA	66 pb	
		SnRK1.2_R	GAGACAGCAAGATAACGAGGGAG		
	**AT3G50500**	*SnRK2.2_F*	ATATGCCATCGGGATCTGAA	115 bp	[4]
		SnRK2.2_R	TTGGTTGGGAATGAAGAACAG		
	**AT5G66880**	*SnRK2.3_F*	GTTGGATGGAAGTCCTGCTC	146 bp	
		SnRK2.3_R	TGCCATCATATTCCTGACGA		
	**AT4G33950**	*SnRK2.6_F*	CACAGGAAGCTTGGACATAGAT	94 pb	[5]
		SnRK2.6_R	GTACACAATCTCTCCGCTACTG		
	**AT1G15330**	*AtPV42a_F*	GGGATTCTCACGATGCTTGAC	135 bp	[6]
		AtPV42a_R	TGTCCAGAGACTGAGTCCTTCG		
	**AT2G43790**	*MAPK6_F*	ACGATGCCATAAGCACCCTTGC	161 bp	[7]
		MAPK6_R	GCGGCTCCATCGCCTCAGAT		
	**AT5G04340**	*ZAT6_F*	AAACCGTGACCTTGACCTGC	300 bp	[8]
		ZAT6_R	CTCCGTTCTTTCCTTCGTAGTG		
	**AT5G59820**	*ZAT12_F*	GAGTCACAAGAAGCCTAACAACGA	242 bp	[9]
		ZAT12_R	AAGCCACTCTCTTCCCACTGCTA		
	**AT5G15410**	*CNGC2_F*	TCTTCAGGTGGATTGGACTGT	89 bp	[10]
		CNGC2_R	TCCACCGTTGATTTGGAGGT		
	**AT5G47910**	*RBOHD_F*	CATGCGGGTGCCCATTT	51 bp	[2]
		RBOHD_R	ATCCGCGGCAATTAAACG		
	**AT1G64060**	*RBOHF_F*	CTTGGCATTGGTGCAACTCC	151 bp	[11]
		RBOHF_R	TCTTTCGTCTTGGCGTGTCA		
	**AT3G18780**	*ACT2_F*	ACTTTCATCAGCCGTTTTGA	190 bp	[12]
		ACT2_R	ACGATTGGTTGAATATCATCAG		

Method S2. High-performance liquid chromatography (HPLC) analysis of 2 mM solutions of Ap_4A and Cp_4C after 2 h leaf epidermal peel treatment.

Potential degradation of Ap_4A and Cp_4C after 2 h incubation on a slide with a leaf was tested. Samples were analysed for purine nucleotides according to [13] with minor modifications. Samples of Ap_4A solution (20 µl) were diluted three times with K_2/KH_2PO_4 buffer and filtered (Anopore 0.2 µm), and analysed by HPLC in the UV–VIS range using a Discovery C18 column (4.6 x 250 mm, 5 µm; Supelco); flow rate 1 ml·min^{-1}. Gradient elution was performed with 0.1 M KH_2PO_4, pH 6.0 (solvent A); solvent A/methanol (9: 1, v/v) (solvent B): 0–9 min, 0% B; 9–15 min, 25% B; 15–17.5 min, 90% B; 17.5–19 min, 100% B; 19–23 min, 100% B and 23–30 min, 0% B. Solutions of pyrimidine nucleotides, Cp_4C, were analysed according to [14] with minor modifications. The samples were chilled, diluted three times with 50 mM TEAB (triethylamine buffer, pH 7.4), and filtered (Anopore 0.2 µm). Then, samples were analysed by HPLC in the UV–VIS range using a Discovery C18 column (4.6 x 250 mm, 5 µm; Supelco); flow rate 1 ml·min^{-1}. The column was eluted with a linear gradient of 50 mM TEAB (pH 7.4) (solvent A) and solvent A:acetonitrile (60:40, v/v) (solvent B); 0–19 min, 40% B. Nucleotides were identified (purines at 260 nm and pyrimidines at 271 nm) and quantified by comparison with respective standards.

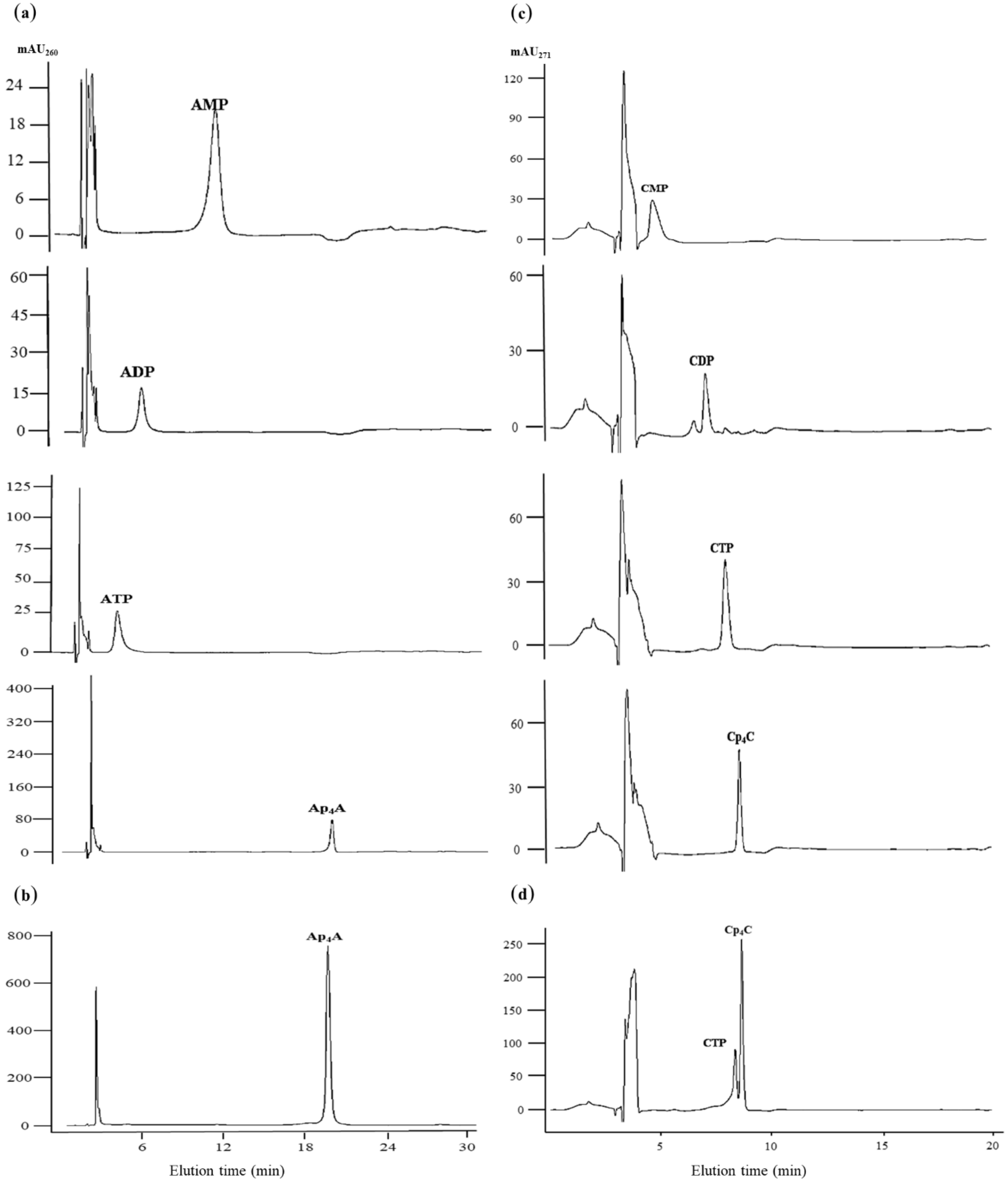

Figure S1. High-performance liquid chromatography analysis of Ap4A and Cp4C solutions after 2 h of leaf epidermal peel treatment. High-performance liquid chromatography (HPLC) analysis was carried out to elucidate Ap4A and Cp4C potential degradation during 2 h of treatment. Samples were analysed for purine nucleotides according to [13], and samples were analysed for pyrimidine nucleotides according to [14]. (**a**) Chromatography of adenine (AMP, ADP, ATP and Ap4A) and (**c**) cytidine (CMP, CDP, CTP and Cp4C) nucleotide standards, and (**b**) Ap4A and (**d**) Cp4C solution from microscope slide after 2 h of epidermal peel treatment. As we observed, a small amount of CTP was detected only in the sample containing Cp4C.

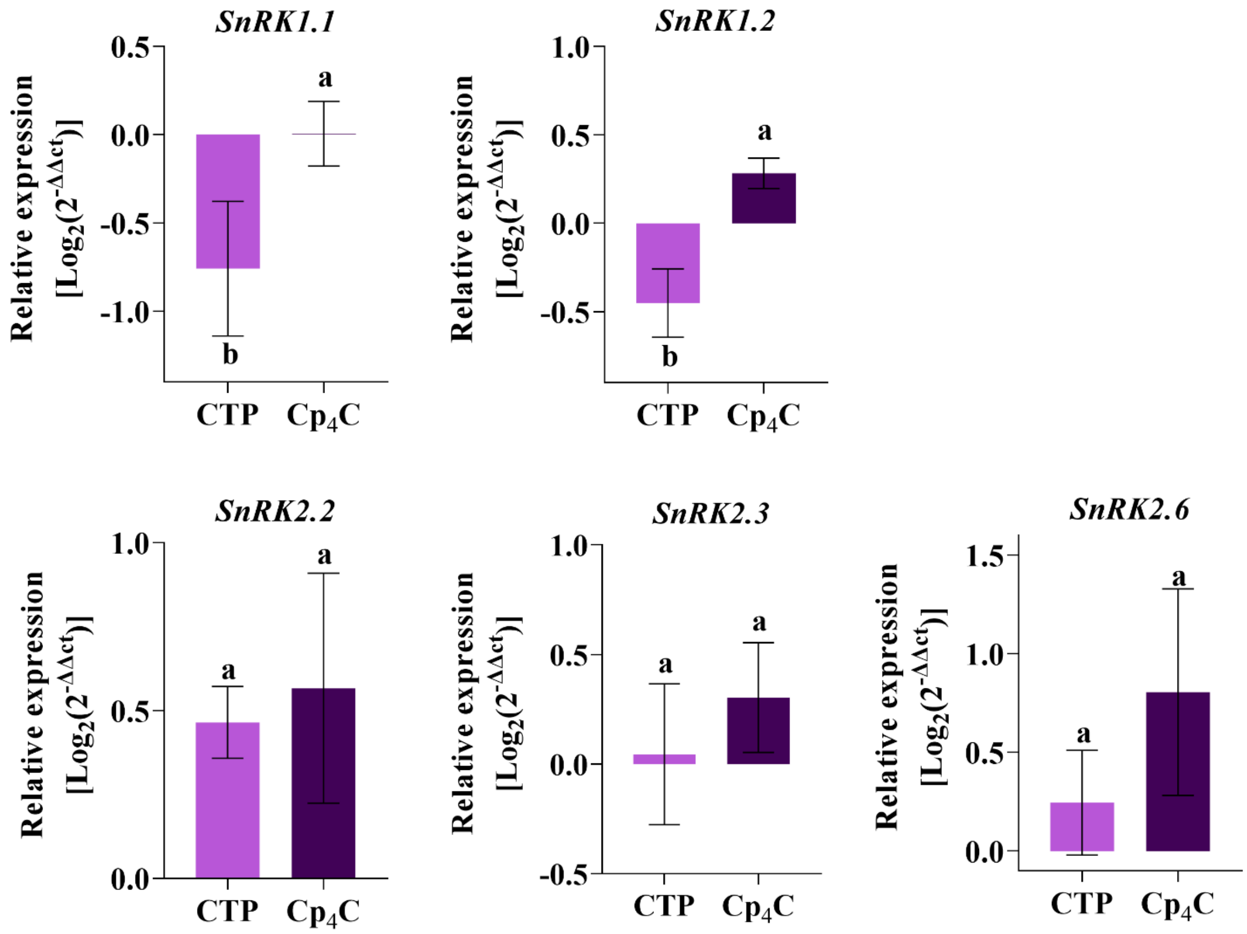

Figure S2. CTP and Cp$_4$C do not up-regulate expression of *SnRK* genes**.** Relative gene expression in 4-week-old leaves of Col-0 and *dorn1*-3 treated with a 2 mM solution of CTP and Cp$_4$C for 2 h. Afterwards, the total RNA was isolated from leaves and transcribed into cDNA, which was used as a template for quantitative real-time PCR, according to the description in the Material and Methods. Transcript expression is represented as the $Log_2(2^{-\Delta\Delta Ct})$ compared to MOCK-treated plants. The housekeeping gene *AtACT2* was used for data normalisation as an endogenous control. Data are mean ± SD from three independent trials within >3 biological replicates. According to the ANOVA statistical analysis and Tukey's HSD multiple range test ($p < 0.05$), values with different letters above the error bars are statistically significant.

References

1. Doyle, J.L.; Doyle J.M. A Rapid DNA Isolation Procedure for Small Quantities of Fresh Leaf Tissue. *PHYTOCHEMICAL BULLETIN* **1987**, *19*, 11–15.
2. Choi, J.; Tanaka, K.; Cao, Y.; Qi, Y.; Qiu, J.; Liang, Y.; Lee, S.Y.; Stacey, G. Identification of a Plant Receptor for Extracellular ATP. *Science* **2014**, *343*, 290–294, doi:10.1126/science.343.6168.290.
3. Sun, D.; Fang, X.; Xiao, C.; Ma, Z.; Huang, X.; Su, J.; Li, J.; Wang, J.; Wang, S.; Luan, S.; et al. Kinase SnRK1.1 Regulates Nitrate Channel SLAH3 Engaged in Nitrate-Dependent Alleviation of Ammonium Toxicity. *Plant Physiology* **2021**, *186*, 731–749, doi:10.1093/plphys/kiab057.

4. Nakashima, K.; Fujita, Y.; Kanamori, N.; Katagiri, T.; Umezawa, T.; Kidokoro, S.; Maruyama, K.; Yoshida, T.; Ishiyama, K.; Kobayashi, M.; et al. Three *Arabidopsis* SnRK2 Protein Kinases, SRK2D/SnRK2.2, SRK2E/SnRK2.6/OST1 and SRK2I/SnRK2.3, Involved in ABA Signaling Are Essential for the Control of Seed Development and Dormancy. *Plant and Cell Physiology* **2009**, *50*, 1345–1363, doi:10.1093/pcp/pcp083.
5. Ou, X.; Li, T.; Zhao, Y.; Chang, Y.; Wu, L.; Chen, G.; Day, B.; Jiang, K. Calcium-Dependent ABA Signaling Functions in Stomatal Immunity by Regulating Rapid SA Responses in Guard Cells. *Journal of Plant Physiology* **2022**, *268*, 153585, doi:10.1016/j.jplph.2021.153585.
6. Fang, L.; Hou, X.; Lee, L.Y.C.; Liu, L.; Yan, X.; Yu, H. AtPV42a and AtPV42b Redundantly Regulate Reproductive Development in *Arabidopsis Thaliana*. *PLoS ONE* **2011**, *6*, e19033, doi:10.1371/journal.pone.0019033.
7. Kannan, P.; Pandey, D.; Gupta, A.K.; Punetha, H.; Taj, G.; Kumar, A. Expression Analysis of MAP2K9 and MAPK6 during Pathogenesis of *Alternaria Blight* in *Arabidopsis thaliana* Ecotype Columbia. *Mol Biol Rep* **2012**, *39*, 4439–4444, doi:10.1007/s11033-011-1232-1.
8. Shi, H.; Liu, G.; Wei, Y.; Chan, Z. The Zinc-Finger Transcription Factor ZAT6 Is Essential for Hydrogen Peroxide Induction of Anthocyanin Synthesis in *Arabidopsis*. *Plant Mol Biol* **2018**, *97*, 165–176, doi:10.1007/s11103-018-0730-0.
9. Le, C.T.T.; Brumbarova, T.; Ivanov, R.; Stoof, C.; Weber, E.; Mohrbacher, J.; Fink-Straube, C.; Bauer, P. ZINC FINGER OF ARABIDOPSIS THALIANA12 (ZAT12) Interacts with FER-LIKE IRON DEFICIENCY-INDUCED TRANSCRIPTION FACTOR (FIT) Linking Iron Deficiency and Oxidative Stress Responses. *Plant Physiol* **2016**, *170*, 540–557, doi:10.1104/pp.15.01589.
10. Wang, L.; Ning, Y.; Sun, J.; Wilkins, K.A.; Matthus, E.; McNelly, R.E.; Dark, A.; Rubio, L.; Moeder, W.; Yoshioka, K.; et al. *Arabidopsis Thaliana* CYCLIC NUCLEOTIDE-GATED CHANNEL2 Mediates Extracellular ATP Signal Transduction in Root Epidermis. *New Phytologist* **2022**, *234*, 412–421, doi:10.1111/nph.17987.
11. Morales, J.; Kadota, Y.; Zipfel, C.; Molina, A.; Torres, M.-A. The Arabidopsis NADPH Oxidases *RbohD* and *RbohF* Display Differential Expression Patterns and Contributions during Plant Immunity. *EXBOTJ* **2016**, *67*, 1663–1676, doi:10.1093/jxb/erv558.
12. Pietrowska-Borek, M.; Nuc, K.; Guranowski, A. Exogenous Adenosine 5′-Phosphoramidate Behaves as a Signal Molecule in Plants; It Augments Metabolism of Phenylpropanoids and Salicylic Acid in *Arabidopsis thaliana* Seedlings. *Plant Physiology and Biochemistry* **2015**, *94*, 144–152, doi:10.1016/j.plaphy.2015.05.013.
13. Guranowski, A.; Starzyńska, E.; Pietrowska-Borek, M.; Rejman, D.; Blackburn, G.M. Novel Diadenosine Polyphosphate Analogs with Oxymethylene Bridges Replacing Oxygen in the Polyphosphate Chain: Potential Substrates and/or Inhibitors of Ap4A Hydrolases. *FEBS Journal* **2009**, *276*, 1546–1553.
14. Guranowski, A.; Wojdyła, A.M.; Pietrowska-Borek, M.; Bieganowski, P.; Khurs, E.N.; Cliff, M.J.; Blackburn, G.M.; Błaziak, D.; Stec, W.J. Fhit Proteins Can Also Recognize Substrates Other than Dinucleoside Polyphosphates. *FEBS Letters* **2008**, *582*, 3152–3158.
